# 3D Ultrasound versus Computed Tomography for Tumor Volume Measurement Compared to Gross Pathology—A Pilot Study on an Animal Model

**DOI:** 10.3390/jimaging8120329

**Published:** 2022-12-19

**Authors:** Fatemeh Makouei, Caroline Ewertsen, Tina Klitmøller Agander, Mikkel Vestergaard Olesen, Bente Pakkenberg, Tobias Todsen

**Affiliations:** 1 Department of Otorhinolaryngology, Head and Neck Surgery and Audiology, Rigshospitalet, Copenhagen University Hospital, DK-2100 Copenhagen, Denmark; 2 Department of Radiology, Rigshospitalet, Copenhagen University Hospital, DK-2100 Copenhagen, Denmark; 3 Institute of Clinical Medicine, Faculty of Health and Medical Sciences, University of Copenhagen, DK-2200 Copenhagen, Denmark; 4 Department of Pathology, Rigshospitalet, Copenhagen University Hospital, DK-2100 Copenhagen, Denmark; 5 Centre for Neuroscience and Stereology, Department of Neurology, Bispebjerg-Frederiksberg Hospital, DK-2400 Copenhagen, Denmark; 6 Copenhagen Academy for Medical Education and Simulation, DK-2100 Copenhagen, Denmark

**Keywords:** 3D ultrasound imaging, ex vivo volume analysis, computed tomography, animal model, tumor volume

## Abstract

The margin of the removed tumor in cancer surgery has an important influence on survival. Adjuvant treatments, prognostic complications, and financial costs are required when the pathologist observes a close/positive surgical margin. Ex vivo imaging of resected cancer tissue has been suggested for margin assessment, but traditional cross-sectional imaging is not optimal in a surgical setting. Instead, three-dimensional (3D) ultrasound is a portable, high-resolution, and low-cost method to use in the operation room. In this study, we aimed to investigate the accuracy of 3D ultrasound versus computed tomography (CT) to measure the tumor volume in an animal model compared to gross pathology assessment. The specimen was formalin fixated before systematic slicing. A slice-by-slice area measurement was performed to compare the accuracy of the 3D ultrasound and CT techniques. The tumor volume measured by pathological assessment was 980.2 mm^3^. The measured volume using CT was 890.4 ± 90 mm^3^, and the volume using 3D ultrasound was 924.2 ± 96 mm^3^. The correlation coefficient for CT was 0.91 and that for 3D ultrasound was 0.96. Three-dimensional ultrasound is a feasible and accurate modality to measure the tumor volume in an animal model. The accuracy of tumor delineation on CT depends on the soft tissue contrast.

## 1. Introduction

Successful surgical cancer treatment requires radical surgical resection of the malignant tumor. Positive or close pathology tumor margins will significantly decrease survival and necessitate adjuvant treatments, increasing comorbidity risk and decreasing quality of life. Ex vivo imaging of the resected cancer tissue could, therefore, be useful for the cancer surgeon to assess the tumor margin immediately perioperatively. Different modalities to scan the ex vivo specimen have been proposed, such as magnetic resonance imaging (MRI) [1,2,3], computed tomography (CT) [3,4], and ultrasound [3,5].

CT is an imaging modality that can provide 3D images of the sample, enabling the margin assessment in all directions. Intraoperative imaging of the surgical specimen using CT has been previously proposed as a tool to reduce the number of reoperations [6]. High-resolution images of the ex vivo tissue have been obtained by micro-CT, which enabled the discrimination of cancer area from the normal tissue [7,8].

Ultrasound is a portable and cheap imaging modality that can be used to provide high-resolution visualization of surgical specimens [9]. It is possible to image the border between the tumor and healthy tissue accurately using ultrasound [10,11,12]. The limitation with 2D ultrasound is the user-dependency and generation of dynamic, but only two-dimensional, image “slices” of the tissue. However, new technology improvements have made it possible to obtain immediate 3D ultrasound imaging of the tumor, which, therefore, overcome some of the problems with B-mode ultrasound [13]. While pathology slicing provides interpretations based on the 2D observations, a 3D imaging model holds the potential of a better 3D description of the tumor. Several previous studies have reported a higher diagnostic accuracy using 3D ultrasound compared to the conventional 2D B-mode imaging [3,14,15,16,17].

In this work, we investigate the accuracy of 3D ultrasound, as a portable and low-cost modality, compared to CT to evaluate the tumor volume in an animal specimen.

## 2. Materials and Methods

We conducted an experimental study to compare the tumor volume measurement obtained using 3D ultrasound and CT to gross pathology. We used an animal model to imitate a surgical specimen with a soft tissue tumor. The animal model was made from a small chicken particle (tumor) placed in a piece of calf liver (normal tissue) with dimensions of about 1.5 cm × 2 cm × 3.5 cm. The phantom was wrapped tightly using cling wrap and kept in the fridge overnight. Then, the sample was placed in formalin for 24 h to fixate. The specimen was scanned using 3D ultrasound and CT modalities before it was sliced and evaluated by a pathologist. The tumor and normal tissue had very similar densities.

### 2.1. 3D Ultrasound and Computed Tomography Imaging

**Figure 1:**
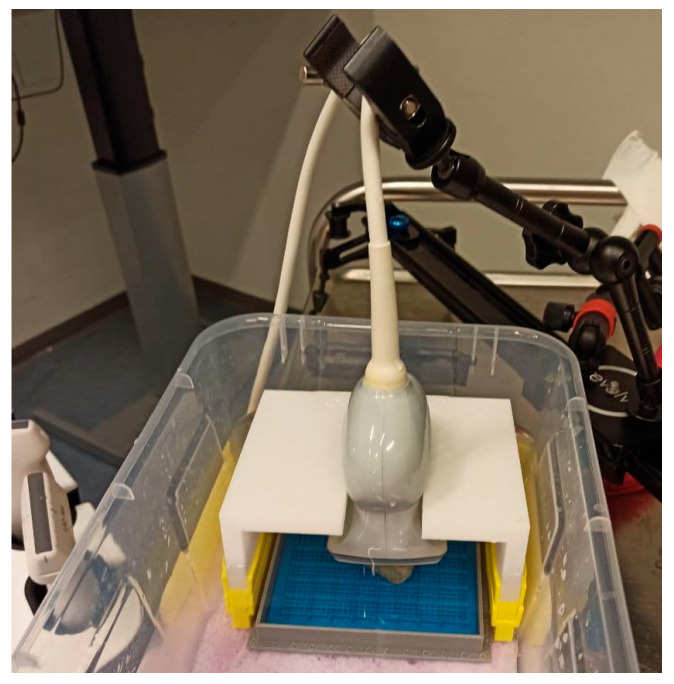
Three-dimensional ultrasound imaging of the specimen. A holder is used to keep the probe at the desired position. The height of the probe front-face in relation to the specimen surface is adjusted using the yellow elongators.

Ultrasound 3D imaging was performed using a SAMSUNG RS85 Prestige ultrasound machine, Copenhagen, Denmark, and a 3D linear ultrasound probe (LV3-14A) with a center frequency of 6.8 MHz. The specimen was placed in a box filled with isotone saline to perform the 3D ultrasound scan. A probe holder and a pair of elongators were designed and 3D-printed to keep the probe at the desired position and depth related to the specimen (see Figure 1).

The depth adjustment was optimized to preserve the highest possible resolution. To detect the curved edges in the axial direction, the compounding feature of the machine was employed. The image was optimized by gain adjustment and by setting the focus point at the center of the specimen.

The animal specimen was scanned with the CT scanner (Canon medical systems, Aquilion ONE, Copenhagen, Denmark) set at 120 kVp, 100 mA, and 0.5 mm of voxel dimension. The images were reconstructed with a 0.5 mm slice thickness and exported as DICOM file series.

### 2.2. Specimen Slicing

**Figure 2:**
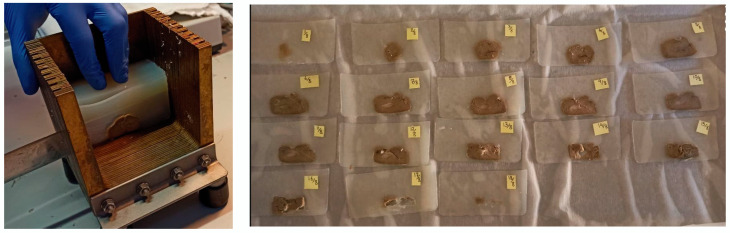
Slicing of the specimen using a specific device that allows for thin and parallel cutting.

To validate the measurements performed by the ultrasound and CT data, a correlation with the gross pathology results was necessary. The formalin-fixated sample was embedded in 4% agar and sliced systematically into 2.0 mm thick slaps with a random starting point within the slab thickness (see Figure 2). Then, all the slaps were photographed for estimating the tumor area of each slide as well as the entire volume of the tumor in the specimen.

The boundaries of the tumor were delineated on the digital images of the slices by a pathologist. The pathological assessment was considered as the gold standard and used to evaluate the results from 3D ultrasound and CT.

### 2.3. Three-Dimensional Ultrasound and CT Data Processing

The acquired volumetric ultrasound images and CT scan data were imported in ITK-SNAP segmentation software, which is a free software application used to segment structures in 3D medical images (www.itksnap.org (accessed on 10 February 2022)) [18]. A consultant in diagnostic radiology delineated the tumor three times in both 3D ultrasound and CT images blinded to the final gross pathology findings. Delineation was performed at every third plane, which is within ITK-SNAP application recommendation. Then, the labels were interpolated to fill in sparsely drawn segmentations. The contrast adjustment was performed to visualize the soft tissue in CT data. Then, the segmentation was exported as MetaImage data and imported in ParaView (www.paraview.org (accessed on 20 February 2022)) for plane-by-plane area calculation [19]. A pipeline was designed in ParaView, which can make parallel slices in the CT and ultrasound image volume at equal distances in the desired direction. Then, an integration filter was defined to calculate the area of the region of interest at each plane.

Tumor area measurement on gross pathology was performed by an integration function in MATLAB (www.mathworks.com (accessed on 20 February 2022)) [20]. The mean ($A_{mean}$) and standard deviation (*S*) of the results were calculated by Equations (1) and (2), respectively.

(1) $$A_{mean}\left(n\right)=\frac{1}{L}\sum_{l=1}^{L}A_{l}\left(n\right)$$

(2) $$S\left(n\right)=\sqrt{\frac{1}{L-1}\sum_{l=1}^{L}{\left|A_{l}\left(n\right)-A_{mean}\left(n\right)\right|}^{2}.}$$

where *n* is the slice number, *L* is the number of the tumor delineation rounds, and $A_{l}\left(n\right)$ is the measured area of slice number *n* at the *l*th delineation session.

Tumor volume ($V_{t}$) was calculated by multiplication of the summation of the mean slice areas ($A_{mean})$ by the slice intervals (*d*), which is 2 mm in this work.

(3) $$V_{t}=d\times \sum_{n=1}^{N}A_{mean}\left(n\right)$$

The error percentage in the volume measurement could be calculated using the following equation:

(4) $$E=100\times \left(1-\frac{V_{t}}{V_{gp}}\right)\;$$

where $V_{gp}$ is the gold-standard gross pathology volume.

We also measured the root-mean-square error (*RMSE*) of the results by the following equation:

(5) $$RMSE=\sqrt{\frac{1}{N}\sum_{n=1}^{N}{\left|A_{mean}\left(n\right)-A_{gp}\left(n\right)\right|}^{2}.}$$

where $A_{gp}\left(n\right)$ is the gross pathology tumor area measurement on the *n*th slide.

## 3. Results

**Figure 3:**
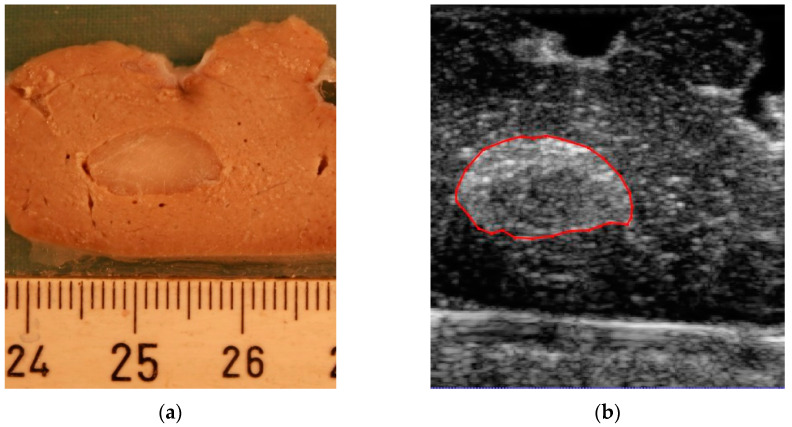

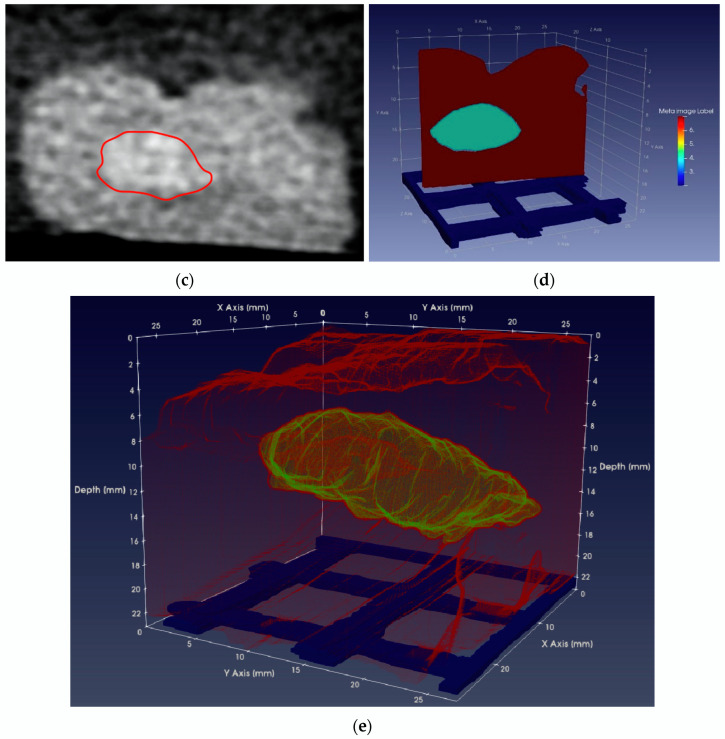
An example of the correlation between slices for gross pathology assessment and corresponding imaging. (**a**) Specimen slice, (**b**) corresponding slice on 3D ultrasound image, (**c**) corresponding slice on CT image, (**d**) corresponding slice on segmentation, and (**e**) 3D segmentation of the animal model.

By the slicing technique described above, the model was sliced into 18 parallel 2 mm thick sections. A total of 11 of the slices included the tumor. When slicing the model, two of the slices were damaged, so we did not include them in the experiment. An example of the correlation between slices for gross pathology assessment and the corresponding 3D ultrasound and CT is presented in Figure 3. Figure 3a shows the digital image from a 2 mm thick slice. The corresponding planes from the 3D ultrasound volume and CT are presented in Figure 3b,c, respectively. Figure 3d shows the segmentation result in which two different labels are allocated to the tumor and surrounding healthy tissue region. A 3D image of the segmentation result is presented in Figure 3e.

**Figure 4:**
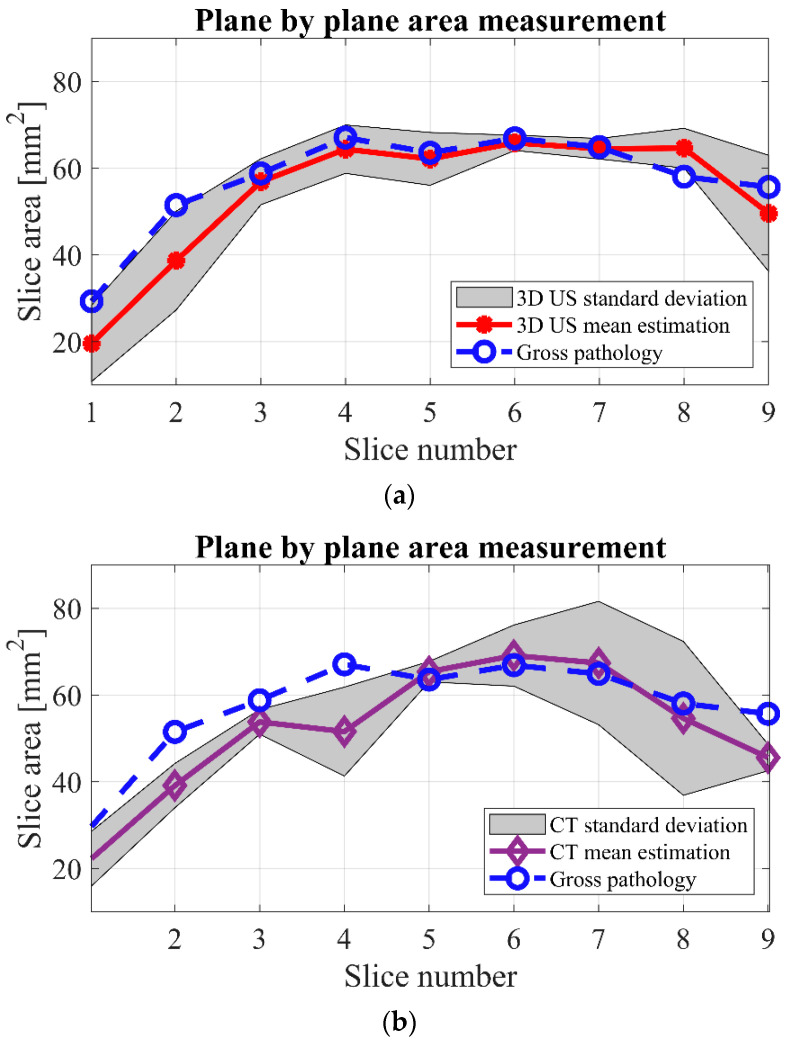
Area measurement at parallel equally distanced planes corresponding to the pathological slices. The dashed blue line is the result of the pathological assessment, and the shaded gray area is the standard deviation of the three measurements. (**a**) 3D ultrasound results compared to the pathological assessment. The solid red line is the mean area at each slice obtained by 3D ultrasound. (**b**) CT results compared to the pathological assessment. The solid purple line is the mean area at each slice measured by CT.

The mean tumor area (Equation (1)) from the CT and 3D ultrasound at parallel, equally distanced planes corresponding to the pathological slices were measured and are reported in Figure 4 and Table 1.

The tumor volume (Equation (3)) measured by gross pathological findings was 980.2 mm^3^. The measured volume using CT was 890.4 ± 90 mm^3^, and 3D ultrasound resulted in a tumor volume of 924.2 ± 96 mm^3^. By using Equation (4), the volume measurement errors using CT and 3D ultrasound were 9.1% and 5.7%, respectively. To investigate how close the area measurements by 3D ultrasound/CT are to the gross pathology areas, we used Equation (5) for RMSE calculation. RMSE was 6.3 for the area measurements from 3D ultrasound and 8.2 for the results from CT.

According to the results presented in Figure 4, a higher overlap between the standard deviation of the 3D ultrasound measurements and the gross pathology was observed compared to the results from CT.

**Figure 5:**
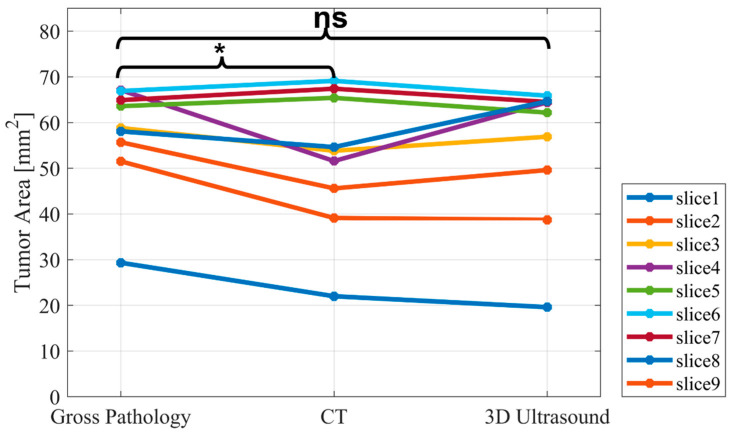
Paired *t*-test for statistical evaluation of the CT and 3D ultrasound compared to gross pathology. ns stands for non-significant, and * stands for significant.

To statistically validate the hypothesis against measured data, we applied the paired *t*-test to test for significant differences between gross pathology and CT/3D ultrasound. As seen in Figure 5, the *p*-value for CT and 3D ultrasound was 0.045 and 0.121, respectively, showing a difference between gross pathology and CT, but not 3D ultrasound. However, the *p*-value for CT was still very close to 0.05. The correlation coefficient for CT was 0.91 and that for the 3D ultrasound was 0.96.

## 4. Discussion

In this experimental study, we found that 3D ultrasound is a feasible and accurate imaging method to assess the tumor volume in an animal specimen. The method is comparable to CT. The results were correlated to the gross pathological findings, which were considered the gold standard. An almost consistent standard deviation was observed in the results of 3D ultrasound (Figure 4a), with a higher variation present in the results obtained by CT (Figure 4b). These results suggest higher predictability of the accuracy of the results obtained from 3D ultrasound compared to CT. In addition, the standard deviation of the 3D ultrasound technique covered most of the data from gross pathology, while this was not the case in CT. Together, this points to the possibility of obtaining results closer to the pathological assessment by the ultrasound method compared to CT. Another advantage was the higher resolution of the ultrasound in the imaging of superficial soft tissue compared to CT, as can be seen in Figure 3b,c. The higher resolution in ultrasound volume led to a higher differentiation between the tumor and healthy tissue. Therefore, it was easier to delineate the tumor borders.

The strength of the current experiment is the precise slicing technique. The slicing device provided equal thickness and similar cutting directions for all the slices. This significantly reduces the error due to miscorrelation of the equal slices in different methods.

Our study finds that 3D ultrasound could be a promising modality to evaluate surgical specimens. The low cost and portability of ultrasound make it very suitable to implement in the operation room, while the specimen would need to be taken to the radiology department to be scanned by CT. Therefore, further studies are needed to explore the possibility of transferring the findings from current work to surgical specimen margin analysis.

### Limitations

In this work, we used an animal model to conduct a proof-of-concept experiment to examine if 3D ultrasound leads to results comparable to CT when correlating to the gold standard of gross pathology. However, we do not know if the animal model is comparable to a real cancer model where the tumor cells are invading normal tissue. Moreover, we have only analyzed one animal specimen and the results, as well as discussions, are inferred from a limited experiment. Therefore, we are not able to compare the variability between different cases of tumors.

The three-dimensional ultrasound scan by the technique employed in the current work suffers from a limited field of view (37.4 mm × 29°), which could be a challenge for a larger specimen. One solution to this challenge could be freehand 3D ultrasound techniques. Freehand scanners enable the examination of the region of interest (ROI) in arbitrary directions and positions. However, positions and orientations of 2D B-scans are required for reconstructing 3D images, which adds up to the complexity of the method, and the accuracy of the results needs to be explored further.

Our animal model had the disadvantage that the density of the “tumor-tissue” and the background tissue was very similar, resulting in very low contrast between the tissues on CT. In patients, this is overcome by including a contrast agent in the examination, but this is not feasible ex vivo. The delineation of the tumor would probably have been more accurate with a larger difference in densities.

## 5. Conclusions

Three-dimensional ultrasound is a feasible imaging method for the measurement of soft tissue volume in an animal model comparable to CT. However, this is a pilot study of the concept and further studies on animal models as well as clinical trials on different tumor types are necessary to confirm the result. The low cost and portability of ultrasound make this a promising imaging modality that may be useful in the operating room for tumor assessment.

## Figures and Tables

**Table 1:** Mean area (*A_mean* ± *S*) in mm^2^ measured on each slice by CT, 3D ultrasound, and gross pathology. The mean results are calculated by averaging over three different delineation sessions.

Slice Number	CT	3D Ultrasound	Gross Pathology
1	22.0 ± 6.3	19.6 ± 8.8	29.3
2	39.1 ± 5.1	38.7 ± 11.4	51.5
3	53.8 ± 2.8	56.9 ± 5.3	58.8
4	51.6 ± 10.2	64.4 ± 5.6	67.1
5	65.4 ± 2.3	62.2 ± 6.0	63.6
6	69.1 ± 7.0	65.9 ± 1.7	66.9
7	67.4 ± 14.2	64.5 ± 2.4	64.9
8	54.6 ± 17.7	64.6 ± 4.6	58.1
9	45.6 ± 3.0	49.6 ± 13.4	55.7

## Data Availability

The experimental data in this study are available per request.
